# XPC inhibits NSCLC cell proliferation and migration by enhancing E-Cadherin expression

**DOI:** 10.18632/oncotarget.3542

**Published:** 2015-03-12

**Authors:** Tiantian Cui, Amit Kumar Srivastava, Chunhua Han, Linlin Yang, Ran Zhao, Ning Zou, Meihua Qu, Wenrui Duan, Xiaoli Zhang, Qi-En Wang

**Affiliations:** ^1^ Department of Radiology, The Ohio State University Wexner Medical Center, Columbus, OH, USA; ^2^ Department of Radiation Oncology, The Ohio State University Wexner Medical Center, Columbus, OH, USA; ^3^ Department of Internal Medicine, The Ohio State University Wexner Medical Center, Columbus, OH, USA; ^4^ Center for Biostatistics, The Ohio State University Wexner Medical Center, Columbus, OH, USA

**Keywords:** XPC, E-Cadherin, non-small-cell lung cancer, Snail, ERK pathway

## Abstract

Xeroderma pigmentosum complementation group C (XPC) protein is an important DNA damage recognition factor in nucleotide excision repair. Deletion of XPC is associated with early stages of human lung carcinogenesis, and reduced XPC mRNA levels predict poor patient outcome for non-small cell lung cancer (NSCLC). However, the mechanisms linking loss of XPC expression and poor prognosis in lung cancer are still unclear. Here, we report evidence that XPC silencing drives proliferation and migration of NSCLC cells by down-regulating E-Cadherin. XPC knockdown enhanced proliferation and migration while decreasing E-Cadherin expression in NSCLC cells with an epithelial phenotype. Restoration of E-Cadherin in these cells suppressed XPC knockdown-induced cell growth both *in vitro* and *in vivo*. Mechanistic studies showed that the loss of XPC repressed E-Cadherin expression by activating the ERK pathway and upregulating Snail expression. Our findings indicate that XPC silencing-induced reduction of E-Cadherin expression contributes, at least in part, to the poor outcome of NSCLC patients with low XPC expression.

## INTRODUCTION

Lung cancer is the leading cause of cancer death worldwide. It is estimated that 221,210 new cases of lung and bronchial cancer will be diagnosed in 2015, and 158,040 deaths are estimated to occur [1]. Despite intensive research over the past decades, the 5-year survival of patients with lung cancer is only 16.6% [2]. Like other cancers, lung carcinogenesis is a chronic and a multiple-step process in which accumulation of genetic and epigenetic alternations are involved. The most devastating aspect of lung cancer is metastasis, a process whose underlying mechanisms remain poorly understood. To achieve a more effective treatment of lung cancer, understanding the mechanisms that drive lung cancer progression is essential.

The xeroderma pigmentosum complementation group C (XPC) protein is a DNA repair factor belonging to nucleotide excision repair (NER) [3]. Besides its well-known role in NER, XPC has also been suggested to play a role in the repair of DNA double-strand breaks (DSB) [4] and base excision repair (BER) [5]. XPC knockout mice are highly predisposed to UV radiation-induced skin cancer [6, 7], as well as 2-acetylaminofluorene-induced liver and lung cancer [8]. In addition, XPC^−/−^ mice also display a significant increase in spontaneous lung tumors [9]. The critical role of XPC in various DNA repair pathways is believed to contribute to the link between the loss of XPC and carcinogenesis of diverse organs. In addition to its role in carcinogenesis, XPC deficiency is also associated with a poor prognosis of various cancer patients [10, 11, 12]. Mechanistically, XPC deficiency could promote tumor metastatic potential through enhancement of matrix metalloproteinase-1 (MMP1) transcription by p53 [13]. XPC silencing is also able to decrease DNA damage-induced apoptosis and renders tumor cells therapy resistance [14].

E-Cadherin belongs to the cadherin family of cell adhesion molecule, playing an important role in the maintenance of epithelial structure. Besides acting as an epithelial marker, E-Cadherin also plays a role in controlling tumor growth and metastasis [15, 16]. Loss of E-Cadherin is frequently correlated with poor prognosis in human cancer and metastasis in mouse model [17, 18]. Recently, a meta-analysis showed that down-regulation of E-Cadherin was associated with poor overall survival and progression-free survival in patients with NSCLC [19]. Reduction of E-Cadherin in cancer through genetic or epigenetic mechanisms has been implicated in the progression and metastasis of malignancies [20, 21]. In this study, we demonstrate that depletion of XPC in NSCLC cells results in the specific downregulation of E-Cadherin expression. The presence of XPC was able to inhibit NSCLC cell growth and metastasis by enhancing E-Cadherin expression. Additionally, the silencing of XPC in NSCLC cells increased endogenous DNA damage. This accumulation of DNA damage activated the ERK pathway, induced expression of Snail, and subsequently downregulated expression of E-Cadherin.

## RESULTS

### XPC expression is positively correlated with the outcome of NSCLC patients

A previous analysis of 126 NSCLC patients has shown that median survival of patients with lower *XPC* mRNA levels was shorter compared with patients with higher *XPC* mRNA levels [22]. To further elucidate the important role of XPC in the survival of NSCLC patients, we analyzed the relationship between the *XPC* mRNA expression level and the survival of NSCLC patients from 1432 lung tumor samples using publicly available datasets (2013 version) (http://kmplot.com/analysis/index.php?p=service&cancer=lung). The Kaplan-Meier analyses demonstrated that higher *XPC* mRNA expression in NSCLC patients is correlated with an improvement of the overall survival (OS), as well as progression-free (FP) survival of patients. These correlations are more pronounced in patients with adenocarcinoma but not squamous cell carcinoma (Supplementary Figures 1A-D). These analyses further confirmed the tumor suppressor role of XPC in NSCLC.

### XPC inhibits the proliferation and migration of NSCLC cells with an epithelial phenotype

**Figure 1:**
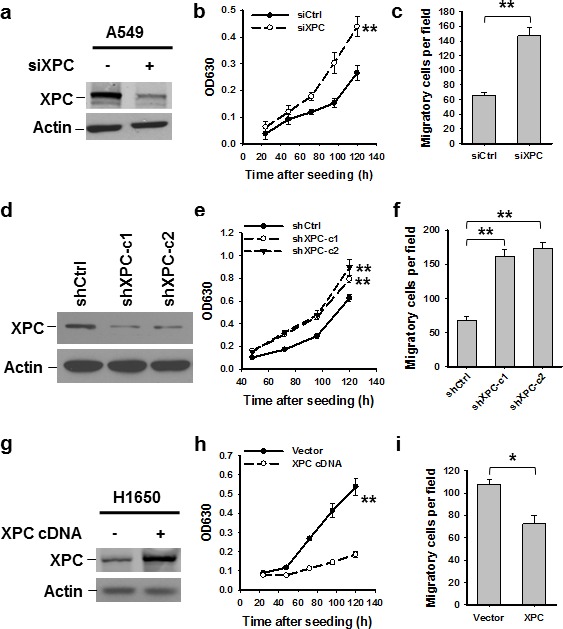
XPC inhibits proliferation and migration of NSCLC cells A549 cells were transiently or stably transfected with siXPC or shXPC, respectively (A-F). H1650 cells were transiently transfected with pcDNA3.1/XPC-V5-His plasmid (G-I). Immunoblotting was conducted to determine the expression of XPC in these cells (A,D,G). Cell growth was measured using the methylene blue assay at various time points. n = 5, bar: SD, **, *P* < 0.01 compared with control siRNA/shRNA/Vector-transfected cells (B,E,H). The transwell migration assay was conducted to quantify the migrated cells. n = 3, bar: SD, **, *P* < 0.01 (C,F,I).

To explore the function of XPC as a tumor suppressor in lung cancer, we first down-regulated XPC expression in NSCLC cell line A549 by transient transfection with XPC siRNA, and analyzed the cell proliferation and migration *in vitro*. As shown in Figures 1A-C, A549 cell proliferation and migration were significantly enhanced after knockdown of XPC. This enhanced cell proliferation and migration capacity was also found in A549 cells with stable XPC knockdown (Figures 1D-F and Supplementary Figures 2A-B). We then overexpressed XPC in another NSCLC cell line H1650, which exhibits a lower expression level of endogenous XPC. Consistent with the result found in A549 cell line, overexpression of XPC was able to inhibit the capacity of cell proliferation and migration in H1650 cells (Figures 1G-I). Surprisingly, we did not find any effect of XPC modulation on the cell growth in H460 cells (Supplementary Figures 3A-B). Given that A549 and H1650 are epithelial cell lines, reflected by the expression of E-Cadherin, while H460 is a mesenchymal cell line without detectable E-Cadherin expression (Supplementary Figures 4A-B), these data indicate that XPC is able to inhibit proliferation and migration of NSCLC cells with an epithelial phenotype.

### XPC enhances E-Cadherin expression in NSCLC cells

**Figure 2:**
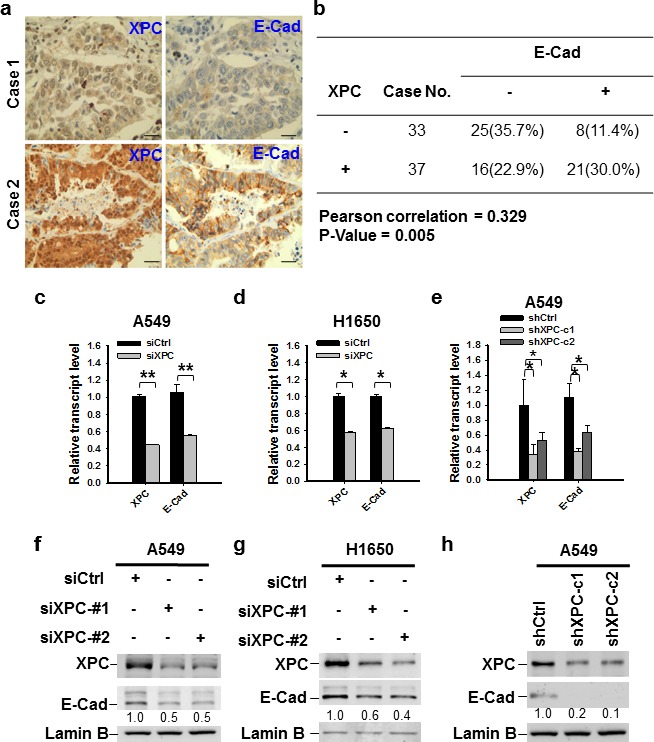
XPC regulates the expression of E-Cadherin in NSCLC cells (A) Paired expression of XPC and E-Cadherin were immunohistochemically examined on the lung cancer tissue microarray (Scale bar: 50 μm). (B) Positive correlation between XPC and E-Cadherin protein expression in human lung tumor tissues (n = 70, *P* = 0.005). Negative and positive expression was defined in the Materials and Methods. (C-E) qRT-PCR was conducted to determine the mRNA expression levels of *XPC* and *E-Cadherin* in A549 and H1650 cells after being transfected with either siRNA or shRNA specific to the human *XPC* gene. n = 3, bar: SD, *, *P* < 0.05; **, *P* < 0.01. (F-H) Immunoblotting analysis was conducted to determine the protein expression of E-Cadherin in A549 and H1650 cells after being transfected with siXPC or shXPC. The intensity of each band was quantified using ImageJ and normalized to Lamin B and then to their corresponding siCtrl/shCtrl-transfected cells.

E-Cadherin is an important cell growth inhibitor [23]. Given that our data indicate XPC regulates cell growth in E-Cadherin expressing cells, we attempted to understand whether XPC regulates the expression of E-Cadherin. Analysis of TCGA data by cBioPortal (http://www.cbioportal.org/public-portal/) demonstrated a positive correlation between *XPC* mRNA expression and E-Cadherin protein expression levels in NSCLC (Supplementary Figure 5). We confirmed this correlation at the protein level by analyzing tissue microarrays that contained 70 lung tumor tissues. Immunohistochemical staining revealed a significant positive correlation between the expression of XPC and E-Cadherin proteins from the same patients (Figures 2A-B). To further investigate the role of XPC in the regulation of E-Cadherin expression, we downregulated XPC expression in A549 and H1650 cells using either siRNA or shRNA specific to the human *XPC* gene, and analyzed the expression of E-Cadherin at both mRNA and protein levels. As shown in Figures 2C-H, knockdown of XPC consistently decreased E-Cadherin expression at both transcript and protein levels, and this positive regulatory role could be confirmed in at least two NSCLC cell lines with siRNA/shRNA targeting different sequences of the *XPC* gene. Taken together, these results indicate that expression of E-Cadherin can be positively regulated by XPC in human NSCLC.

### XPC deficiency promotes NSCLC cell growth through downregulation of E-Cadherin

**Figure 3:**
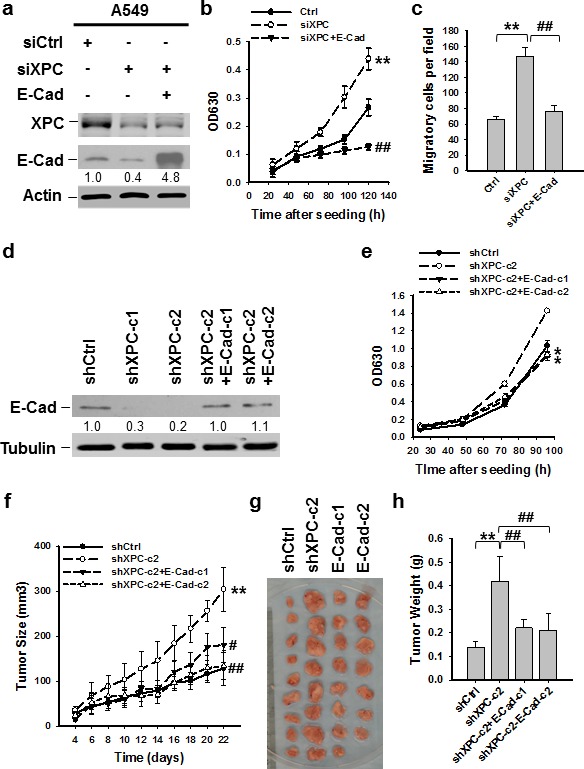
E-Cadherin is able to reverse the effect of XPC downregulation on cell growth (A-C) A549 cells were transiently transfected with siXPC alone, or simultaneously with E-Cadherin expressing vectors. The protein levels of XPC and E-Cadherin were determined using immunoblotting. The intensity of each band was quantified using ImageJ and normalized to Actin and then to their corresponding siCtrl-transfected cells (A). Cell growth was determined using methylene blue staining. n = 5, bar: SD, **, *P* < 0.01 compared with A549-Ctrl cells; ^##^, *P* < 0.01 compared with A549-siXPC cells (B). The transwell migration assay was conducted to quantify the migrated cells. n = 3, bar: SD, **, *P* < 0.01 compared with A549-Ctrl cells; ^##^, *P* < 0.01 compared with A549-siXPC cells (C). (D,E) E-Cadherin expressing vectors were stably transfected into A549-shXPC-c2 cells, and two cell clones were selected (shXPC-c2+E-Cad-c1, and shXPC-c2+E-Cad-c1). The protein level of E-Cadherin was determined using immunoblotting. The intensity of each band was quantified using ImageJ and normalized to Lamin B and then to shCtrl-transfected cells (D). Cell growth was determined using methylene blue staining. n = 5, bar: SD, *, *P* < 0.05 compared with A549-shCtrl cells (E). (F-H) Xenografts were generated by A549 cells (shCtrl), A549 cells with stable XPC knockdown (shXPC-c2), and two clones of A549-shXPC-c2 cells with stable overexpression of E-Cadherin (E-Cad-c1, and E-Cad-c2). Tumor sizes were measured every two days (F). Tumors were removed from mice after 22 days (G), and weighed (H). n = 10, bar: SD, **, *P* < 0.01 compared with A549-shCtrl; ^#^, *P* < 0.05, ^##^, *P* < 0.01 compared with A549-shXPC-c2.

Downregulation of E-Cadherin is regarded as a trigger for cancer invasion and metastasis [24, 16]. Therefore, we sought to determine whether reduced expression of E-Cadherin contributes to XPC deficiency-promoted NSCLC cell proliferation. We transfected siXPC alone or together with E-Cadherin expressing vectors into A549 cells, in which XPC was knocked down, and E-Cadherin was either downregulated, or upregulated (Figure 3A). The siXPC-transfected A549 cells with re-expression of E-Cadherin exhibited decreased cell proliferation and migration compared to those transfected with XPC siRNA alone (Figures 3B-C), indicating that E-Cadherin is able to reverse the effect of XPC downregulation on cell growth. To examine the role of E-Cadherin in XPC-mediated cell growth inhibition *in vivo*, we transfected E-Cadherin into A549 cells that already had stable XPC knockdown (A549-shXPC-c2), generated E-Cadherin stably expressing A549-shXPC cell lines (A549-shXPC-c2-E-Cad-c1 and A549-shXPC-c2-E-Cad-c2) (Figure 3D), and confirmed the function of E-Cadherin overexpression in the inhibition of cell growth in A549-shXPC-c2 cells (Figure 3E). These cells were used to generate xenografts in Athymic nude mice and tumor growth dynamics were recorded. Histological analysis showed typical epithelial cancer cell morphology, and no difference was noted among the xenografts generated by different cell lines (Supplementary Figure 6). Tumor xenografts initiated with A549-shXPC cells grew faster than those derived from XPC-proficient A549-shCtrl cells. However, overexpression of E-Cadherin in A549-shXPC cells significantly retarded the growth of these cells *in vivo* (Figure 3F). The tumor volumes and weights at the end of the experiments also significantly decreased in two E-Cadherin overexpressing groups compared to the A549-shXPC group (Figures 3G-H). These data suggest that E-Cadherin is a downstream effector in the process of XPC-induced inhibition of NSCLC cell proliferation.

### XPC inhibits the expression of Snail in NSCLC cells

**Figure 4:**
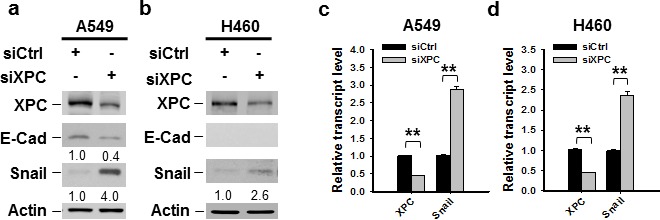
XPC negatively regulates the expression of Snail A549 and H460 cells were transiently transfected with either control or XPC siRNA for 48 h. (A,B) Immunoblotting analysis was conducted to determine the protein levels of XPC, E-Cadherin, Snail and Actin using their corresponding antibodies. The intensity of each band was quantified using ImageJ and normalized to Actin and then to siCtrl-transfected cells. (C,D) qRT-PCR was conducted to determine the mRNA levels of *XPC* and *Snail*. The transcript levels relative to siCtrl-transfected cells were presented. n = 3, bar: SD, **, *P* < 0.01.

One of the mechanisms through which E-Cadherin is downregulated in cancer cells is the transcription repression by Epithelial-mesenchymal transition (EMT)-related transcription factors, such as Snail, Slug and Zeb1 [25, 26, 27, 28]. To determine whether XPC regulates E-Cadherin expression by affecting the expression of these EMT-related transcription factors, we first analyzed the publically available TCGA data by using cBioPortal. The Spearman correlation analyses of the microarray data revealed a significant inverse correlation between *XPC* and *Snai1* (encoding Snail) mRNA levels (Supplementary Figure 7A). However, no significant correlation between *XPC* and *Snail2* (encoding Slug), or *XPC* and *Zeb1* was found (Supplementary Figures 7B-C). We then knocked down the expression of XPC in A549 cells and determined the change of Snail at the protein level. As shown in Figure 4A, downregulation of XPC considerably enhanced Snail expression, concomitant with above-described reduced E-Cadherin expression. The decreased expression of Snail was also observed in two clones of A549 cells with stable XPC knockdown (Supplementary Figure 8). In addition, Snail was also upregulated by XPC silencing in NSCLC cells without E-Cadherin expression (H460 cells) (Figure 4B), suggesting that XPC silencing-induced Snail overexpression is not through E-Cadherin. In line with the immunoblotting results, we also demonstrated an enhanced *Snail* mRNA expression level in both A549 and H460 cells after XPC knockdown (Figures 4C-D). Taken together, these data indicate that XPC may promote E-Cadherin expression by inhibiting the expression of Snail, a transcription repressor of E-Cadherin.

### XPC regulates cell proliferation of NSCLC through the ERK/Snail/E-Cadherin pathway

**Figure 5:**
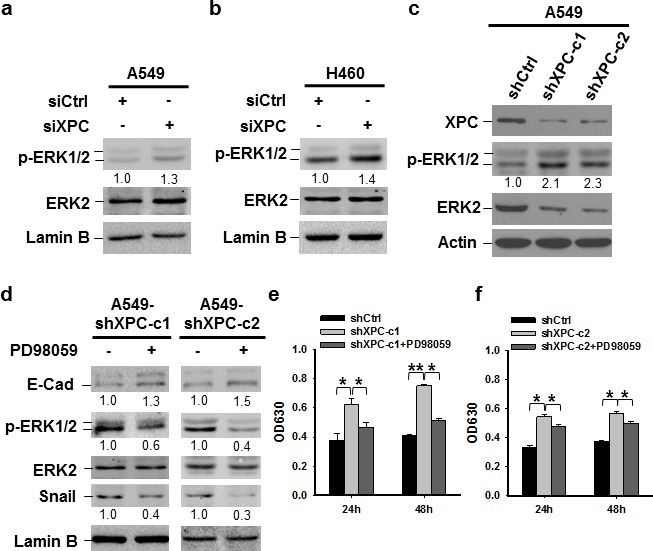
XPC regulates cell proliferation of NSCLC cells through the ERK/Snail/E-Cadherin pathway (A-C) Expression of phospho-ERK1/2 was detected in A549 and H460 cells either transiently transfected with siXPC or stably transfected with shXPC. The intensity of p-ERK1/2 bands was quantified using ImageJ and normalized to ERK2 and then to siCtrl/shCtrl-transfected cells. (D-F) Two clones of A549 cells with stable XPC knockdown were treated with the ERK inhibitor PD98059 for 24 h. Expression of p-ERK1/2, Snail, and E-Cadherin was detected using immunoblotting. The intensity of each band was quantified using ImageJ and normalized to either ERK2, or Lamin B, and then to non-treated cells (D). Cell growth was determined using methylene blue staining. n = 5, bar: SD, *, *P* < 0.05, **, *P* < 0.01 (E,F).

It has been reported that the expression of Snail can be downregulated through the inhibition of the PI3K/AKT or MAPK/ERK pathway [29, 30]. To investigate whether these pathways are affected by XPC modulation, we determined the phosphorylation of AKT and ERK1/2 in A549 and H460 cells after being transiently transfected with XPC siRNA, as well as A549 cells with stable XPC knockdown. We did not see any effect of XPC downregulation on AKT phosphorylation (Supplementary Figure 8). However, downregulation of XPC resulted in an increased phosphorylation of ERK1/2 in both A549 and H460 cell lines (Figures 5A-C), indicating that XPC is able to inhibit the ERK pathway in NSCLC cells. In addition, XPC knockdown-enhanced Snail expression could be blocked by the inhibition of the ERK pathway, accompanied by an increased expression of E-Cadherin (Figure 5D). Furthermore, the ERK pathway inhibition by the ERK1/2 inhibitor could also impede XPC deficiency-induced cell proliferation (Figures 5E-F). These data suggest that XPC insufficiency may promote NSCLC cell growth through the activation of the ERK pathway. Activated ERK pathway enhances Snail expression, which further suppresses E-Cadherin expression, leading to an accelerated cell proliferation.

### Downregulation of XPC increases cellular endogenous DNA damage in NSCLC cells

**Figure 6:**
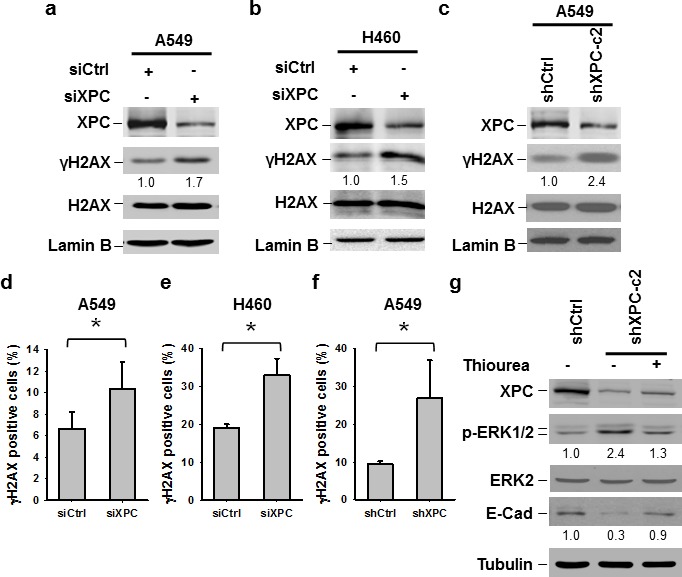
Downregulation of XPC increases endogenous DNA damage (A-C) Immunoblotting analysis was conducted to determine the protein levels of XPC, γH2AX, H2AX and Lamin B using their corresponding antibodies in A549 and H460 cells transiently transfected with either control or XPC siRNA (A,B), and A549 cells stably transfected with shXPC (C). The intensity of each band was quantified using ImageJ and normalized to Lamin B, or H2AX and then to siCtrl/shCtrl-transfected cells. (D-F) Immunofluorescent staining was conducted to visualize γH2AX-positive cells in A549 and H460 cells transiently transfected with either control or XPC siRNA, and A549 cells stably transfected with shXPC. The percenage of γH2AX-positive cells was calculated from at least 3 independnet samples and plotted on the right. Bar: SD *, *P*<0.05. (G) A549-shXPC-c2 cells were treated with the ROS scavenger Thiourea for 24 h. Expression of p-ERK1/2 and E-Cadherin was detected using immunoblotting.

The ERK pathway can be activated by various DNA damages [31]. Given that XPC downregulation is able to elevate the level of intracellular reactive oxygen species (ROS) and results in an accumulation of unrepaired DNA [32], we reasoned that the activation of the ERK pathway in XPC-downregulated NSCLC cells may be due to increased endogenous DNA lesions. Indeed, both A549 and H460 cells with siXPC transfection exhibited elevated amount of γH2AX, an indicator of DNA double-strand breaks (Figures 6A-B). The increased γH2AX could also be detected in A549 cells with stable shXPC transfection (Figure 6C). Immunofluorescent staining further confirmed the increase in γH2AX-positive cells in A549 and H460 cell populations with XPC knockdown (Figure 6D-F, Supplementary Figure 9). To further confirm the role of endogenous ROS-induced DNA damage in the activation of the ERK pathway and downregulation of E-Cadherin in XPC-deficient cells, we treated A549-shXPC cells with ROS scavenger Thiourea. We found that Thiourea treatment reversed the enhancement of ERK1/2 phosphorylation and the reduction of E-Cadherin expression level in XPC-knockdown A549 cells (Figure 6G). These data suggest that XPC deficiency compromises the DNA repair capacity in NSCLC cells, leading to an accumulation of endogenous DNA lesions, which activate the ERK pathway, and further reduce E-Cadherin expression.

## DISCUSSION

As a DNA repair factor, XPC plays an important role in preventing carcinogenesis [11, 33]. However, the positive correlation between XPC expression and the outcome of various cancer patients including lung, breast, gastric, and ovarian cancer (http://www.kmplot.com) indicates that XPC may play an additional role in preventing tumor progression. Mechanistically, XPC deficiency has been reported to increase the invasiveness of lung cancer through downregulation of p27 (kip) and upregulation of skp2 and E2F1 [34]. In addition, this group further demonstrated that XPC defects might alter p53 function, resulting in the promotion of tumor aggressiveness via upregulation of MMP1 [13]. In this study, we present a novel mechanism for the favorable role of XPC in controlling the progression and metastasis of NSCLC. Our data indicates that the deficiency of XPC promotes the expression of Snail, which further represses the expression of E-Cadherin to rescue cancer cells from E-Cadherin-mediated proliferation inhibition.

Loss of E-Cadherin expression or function is a common event in cancer [35]. E-Cadherin is known to suppress tumor cell growth and migration in various malignancies [36, 37]. We have demonstrated in this study that restoration of E-Cadherin expression in XPC-silencing NSCLC cells can neutralize XPC deficiency-induced cell proliferation both *in vitro* and *in vivo*. Combined with our finding that XPC modulation only affected epithelial NSCLC cells that exhibit E-Cadherin expression, we propose that E-Cadherin is a key factor in the XPC-mediated inhibition of NSCLC cell proliferation.

In human cancers, E-Cadherin is frequently silenced by transcriptional or translational mechanisms [38, 39, 40]. Repression of E-Cadherin through E-box-binding proteins, such as Snail, Slug, and Zeb1, has been described in many studies [25, 26, 27, 28]. We observed an increase of Snail expression at the mRNA and protein levels and a concomitant decrease of E-cadherin expression after knockdown of XPC in lung cancer cell lines. Interestingly, XPC knockdown enhanced Snail expression in the absence of E-Cadherin in H460 cells. These results indicate that XPC silencing could upregulate expression of Snail, which is the mechanism underlying the downregulation of E-Cadherin in NSCLC cells with XPC knockdown. The analyses of publicly available microarray datasets showing an inverse correlation between *XPC* and *Snai*1 mRNA levels further supports our conclusion that XPC upregulates E-Cadherin expression by decreasing the expression of Snail.

In this study, we also demonstrated that XPC-deficiency upregulates Snail expression by activation of the ERK pathway, as reflected by the findings that XPC knockdown enhanced phosphorylation of ERK1/2, and that the ERK inhibitor blocked XPC knockdown-induced upregulation of Snail. ERK kinases can activate several transcription factors, including Snail, to further regulate the expression of specific genes [41, 42]. Chen et al.[43] reported that the ERK pathway regulates breast cancer cell migration by maintaining Slug expression. In addition, TGFβ-induced activation of ERK5/MAPK could also increase the transcriptional activity of Snail [44]. Thus, it is very likely that the enhanced Snail expression is induced by an activated ERK pathway in XPC-silencing NSCLC cells.

The XPC protein plays a key role in the global genomic NER (GG-NER), which eliminate a wide variety of DNA lesions, including ultraviolet radiation (UVR)-induced cyclobutane pyrimidine dimers (CPD), 6-4 photoproducts (6-4PP), and environmental carcinogen benzo[a]pyrene-induced bulky adducts, as well as chemotherapeutic cisplatin-induced intra-strand crosslinks. In addition, it has also been reported that XPC plays an important role in the base excision repair (BER) pathway to remove oxidative DNA damage [5], and in DNA double-strand break repair [4]. Consistent with these reports, unrepaired DNA damage accumulated in XPC-deficient mice cells may be an important mechanism for high frequency of spontaneous lung tumor observed in XPC^−/−^ mice [11]. Given that DNA damage is able to activate multiple cellular signal pathways, such as the PI3K/AKT pathway and the MAPK/ERK pathway [32, 45], we reason that DNA damage accumulation in NSCLC cells is one of the triggers for the ERK pathway activation following XPC silencing. However, we do not exclude the possibility that XPC directly regulates Snail expression, because XPC has been indicated to regulate gene transcription through directly binding to the promoter regions [14, 46].

**Figure 7:**
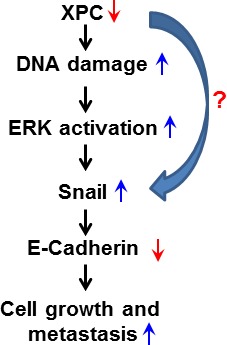
Model of the mechanism by which XPC upregulates E-Cadherin expression and inhibits cell proliferation and metastasis in lung cancer The deficiency of XPC induces the accumulation of the endogenous DNA damage, which activates the ERK pathway. The activated ERK pathway then enhances the expression of Snail, which further represses the expression of E-Cadherin. Decreased expression of E-Cadherin by XPC silencing leads to a promotion of lung cancer cell proliferation and metastasis.

In conclusion, we propose a novel mechanism underlying the favorable role of XPC in the survival of NSCLC patients. As a DNA repair factor, XPC helps to maintain a low level of endogenous DNA lesions in the cancer cells. In the case of XPC deficiency, cellular DNA damage accumulates, activating the ERK pathway. The activated ERK pathway then enhances the expression of Snail, which further represses the expression of E-Cadherin, leading to a promotion of cancer cell proliferation. Alternatively, XPC may also upregulate E-Cadherin expression by directly repressing Snail expression (Figure 7). Therefore, enhancing XPC expression in NSCLC cells with an epithelial phenotype would be a promising strategy to slow down the progression of lung cancer.

## MATERIALS AND METHODS

### Cell culture and treatment

Human lung cancer cell lines A549 and H460 were purchased from ATCC (Manassas, VA). H1650 cells were kindly provided by Dr. Yuan Chen (University Hospital Jena, Germany). Cells were grown in RPMI 1640 medium supplemented with 10% (v/v) fetal bovine serum (FBS), and maintained in a humidified atmosphere with 5% CO_2_ at 37°C. For drug treatment, cells were treated with 50 μM ERK inhibitor PD98059 (LC laboratories, Woburn, MA USA) or 0.1 mM Thiourea (Sigma, Louis, MO, USA) for 24h.

### RNA extraction and quantitative reverse transcription PCR (qRT-PCR)

Total RNA was extracted using Trizol reagent (Life Technologies, Carlsbad, CA), and the first strand cDNA was generated by the Reverse Transcription System (Promega, Madison, WI) in a 20 μl reaction containing 1 μg of total RNA. A 0.5 μl aliquot of cDNA was amplified by Fast SYBR Green PCR Master Mix (Life Technologies) in each 20 μl reaction. PCR reactions were run on the ABI 7900 Fast Real-Time PCR system in the OSUCCC Nucleic Acid Core Facility with the following primers: *XPC*, forward, 5′-GAC AAG CAG GAG AAG GCA AC-3′, reverse, 5′-GGT TCG GAA TCC TCA TCA GA-3′; *E-Cadherin*, forward, 5′-TGC CCA GAA AAT GAA AAA GG-3′, reverse, 5′-GTG TAT GTG GCA ATG CGT TC-3′; *Snail*, forward, 5′-CCT CAA GAT GCA CAT CCG AAG-3′, reverse, 5′-ACA TGG CCT TGT AGC AGC CA-3′; *GAPDH*, forward, 5′-GAA GGT GAA GGT CGG AGT-3′, reverse, 5′-GAA GAT GGT GAT GGG ATT TC-3′. The relative expression values of *XPC*, *E-Cadherin*, and *Snail* were calculated and normalized to *GAPDH* in each sample and compared. The experiments were performed in triplicates.

### Western blot analysis

Whole cell lysates were prepared by boiling cell pellets for 10 min in SDS lysis buffer [2% SDS, 10% Glycerol, 62 mM Tris-HCl, pH 6.8 and a complete mini-protease inhibitor cocktail (Roche Applied Science, Indianapolis, IN)]. After protein quantification with Bio-Rad Dc Protein Assay (Bio-Rad Laboratories, Hercules, CA), equal amounts of proteins were loaded, separated on a polyacrylamide gel, and transferred to a nitrocellulose membrane. Protein bands were immuno-detected with appropriate antibodies, e.g., rabbit-anti-XPC [47], mouse-anti-E-Cadherin (BD Transduction Laboratories, San Jose, CA, Cat. No. 610181, 1:1000), rabbit-anti-Snail (Cell Signaling, Danvers, MA, Cat. No. 3879, 1:1000), rabbit-anti-pERK1/2 (Cell Signaling, Cat. No. 9101, 1:1000), rabbit-anti-ERK2 (Cell Signaling, Cat. No. 9108, 1:1000), mouse-anti-γH2AX (Millipore, Billerica, MA, Cat. No. 05-636, 1:1000), rabbit-anti-H2AX (Cell Signaling, Cat. No. 7631, 1:1000), mouse-anti-Actin (Santa Cruz Biotechnology, Dallas, TX, Cat. No. sc-47778, 1:1000), and goat-anti-Lamin B (Santa Cruz Biotechnology, Cat. No. sc-6216, 1:1000).

### Immunofluorescence

The experiments were conducted according to a method established in our laboratory [47]. The cells were washed twice with cold PBS, and fixed with 2% paraformaldehyde in 0.5% Triton X-100 at 4°C for 30 min. The fixed cells were blocked with 20% normal goat serum, and stained with an appropriate primary antibody (anti-E-Cadherin, 1:100; anti-γH2AX, 1:200), followed by Fluor488-, or Fluor594-conjugated secondary antibody. The coverslips were mounted in Vectashield mounting medium with DAPI. The fluorescence images were obtained with a Nikon fluorescence microscope (E80i, Tokyo, Japan) and processed with SPOT software.

### Tissue Microarray and Immunohistochemistry (IHC)

Confirmed, formalin-fixed, paraffin-embedded human lung tumor tissue array containing duplicated 70 cases covering all the common types of lung cancer and 5 cases of normal and other non-malignant lung tissues were purchased from BioChain (Newark, CA). IHC was performed as previously described [48]. Primary rabbit antibody against XPC (1:1000) and mouse antibody against E-Cadherin (1:100) were used. Tissues were counterstained with Richard Allen hematoxylin. Intensity of staining was scored semi-quantitatively as negative (score 0), weak (score 1), moderate (score 2) or strong (score 3) as previously described [48]. For statistical evaluation, score 0 and 1 were considered as negative (−), while scores 2 and 3 together were as positive (+).

### Plasmids, siRNA and transfection

pcDNA3.1/XPC-V5-His plasmid containing XPC cDNA was described previously [47]. XPC shRNA (short hairpin RNA) were purchased from Sigma (TRCN0000083118, TRCN0000083119). XPC siRNA and a scramble non-targeting siRNA (siCtrl) were purchased from Dharmacon (siGENOME human XPC siRNA SMARTpool, siXPC-#1: 5′-GCA AAU GGC UUC UAU CGA A-3′, siXPC-#2: 5′-GGA GGG CGA UGA AAC CUU U-3′, siCtrl: 5′-UUC UCC GAA CGU GUC ACG U-3′). pcDNA3/E-Cadherin plasmid containing E-Cadherin cDNA (NM_004360.3) was purchased from Addgene (Addgene plasmid #45769, ref: [49]) The plasmids and siRNA were transfected into cells by using Lipofectamine 2000 reagents (Life Technologies) according to manufacturer's instructions.

### Cell proliferation assay

Cells were seeded in 60-mm dishes at an initial density of 2.5× 10^5^ cells/dish, siRNA transfection was performed on the second day. After 24 hs, cells were trypsinized and seeded in 96-well plates at an initial density of 500 cells/well. On the following days, the cells were washed with PBS, fixed with 3.7% formaldehyde for 30 min and stained with 1.0% methylene blue for 30 min. The plate was rinsed in running water and then left to dry. One hundred microliters of solvent (10% acetic acid, 50% methanol and 40% H_2_O) was added to each well to dissolve the cells and optical density of the released color was read at 630 nm.

### Cell migration assay

Cells (2×10^3^/well) were resuspended in 300 μl RPMI1640 medium containing 10% (v/v) FBS, and placed in the upper transwell chamber (8 μm pore size, BD Biosciences). The upper chamber was placed in a 24-well culture dish containing 1000 μl medium. After incubation for 24 h at 37°C, non-migrated cells on the upper membrane were removed with a cotton swab. Migrated cells on the bottom surface were fixed with cold methanol and stained with crystal violet. The migrated cell number was counted. All the experiments were performed in triplicates.

### Xenograft tumor growth

Athymic NCr-nu/nu mice (6-8 weeks, female, 20-25 g body weight) were obtained from Charles River. Animals were maintained in accordance with institutional policies, and all studies were performed with approval of the IACUC at the Ohio State University. To generate xenografts, 5×10^6^ cells were mixed (1:1) with Matrigel (BD Biosciences) and injected subcutaneously into the flank of each mouse. Tumor growth was measured using calipers, and volumes were calculated based on the formula V= (*a* × *b*^2^)/2, in which *a* is the longest and *b* is the shortest diameter of the tumor. At the end of the experiment, the animals were euthanized, and the tumor mass was harvested, weighed and photographed.

### Statistical analysis

Results are presented as the mean ± SD for at least three independent experiments for each group. Chi-square exact test and Spearman correlation were applied to analyze the association between the expression of XPC and E-Cadherin. Statistic differences were determined by using ANOVA or two sample t-tests for independent samples. Linear mixed effects models were used for analysis to take account of correlations among correlated observations, such as the cell growth measured over time in cell culture or in the *in vivo* mouse models. *P* values less than 0.05 were defined as statistically significant after adjustment for multiple comparisons using Holm's procedure.

## SUPPLEMENTARY MATERIALS, FIGURES



## References

[1] Siegel RL, Miller KD, Jemal A (2015). Cancer statistics, 2015. CA Cancer J Clin.

[2] Howlader N, Noone AM, Yu M, Cronin KA (2012). Use of imputed population-based cancer registry data as a method of accounting for missing information: application to estrogen receptor status for breast cancer. Am J Epidemiol.

[3] Legerski R, Peterson C (1992). Expression cloning of a human DNA repair gene involved in xeroderma pigmentosum group C. Nature.

[4] Despras E, Pfeiffer P, Salles B, Calsou P, Kuhfittig-Kulle S, Angulo JF, Biard DS (2007). Long-term XPC silencing reduces DNA double-strand break repair. Cancer Res.

[5] D'Errico M, Parlanti E, Teson M, de Jesus BM, Degan P, Calcagnile A, Jaruga P, Bjoras M, Crescenzi M, Pedrini AM, Egly JM, Zambruno G, Stefanini M, Dizdaroglu M, Dogliotti E (2006). New functions of XPC in the protection of human skin cells from oxidative damage. EMBO J.

[6] Sands AT, Abuin A, Sanchez A, Conti CJ, Bradley A (1995). High susceptibility to ultraviolet-induced carcinogenesis in mice lacking XPC. Nature.

[7] Cheo DL, Meira LB, Hammer RE, Burns DK, Doughty AT, Friedberg EC (1996). Synergistic interactions between XPC and p53 mutations in double-mutant mice: neural tube abnormalities and accelerated UV radiation-induced skin cancer. Curr Biol.

[8] Cheo DL, Burns DK, Meira LB, Houle JF, Friedberg EC (1999). Mutational inactivation of the xeroderma pigmentosum group C gene confers predisposition to 2-acetylaminofluorene-induced liver and lung cancer and to spontaneous testicular cancer in Trp53−/− mice. Cancer Res.

[9] Melis JP, Wijnhoven SW, Beems RB, Roodbergen M, Van den Berg J, Moon H, Friedberg E, Van der Horst GT, Hoeijmakers JH, Vijg J, van SH (2008). Mouse models for xeroderma pigmentosum group A and group C show divergent cancer phenotypes. Cancer Res.

[10] Yang PW, Hsieh CY, Kuo FT, Huang PM, Hsu HH, Kuo SW, Chen JS, Lee JM (2013). The survival impact of XPA and XPC genetic polymorphisms on patients with esophageal squamous cell carcinoma. Ann Surg Oncol.

[11] Hollander MC, Philburn RT, Patterson AD, Velasco-Miguel S, Friedberg EC, Linnoila RI, Fornace AJ (2005). Deletion of XPC leads to lung tumors in mice and is associated with early events in human lung carcinogenesis. Proc Natl Acad Sci U S A.

[12] Yang J, Xu Z, Li J, Zhang R, Zhang G, Ji H, Song B, Chen Z (2010). XPC epigenetic silence coupled with p53 alteration has a significant impact on bladder cancer outcome. J Urol.

[13] Wu YH, Wu TC, Liao JW, Yeh KT, Chen CY, Lee H (2010). p53 dysfunction by xeroderma pigmentosum group C defects enhance lung adenocarcinoma metastasis via increased MMP1 expression. Cancer Res.

[14] Wang QE, Han C, Zhang B, Sabapathy K, Wani AA (2012). Nucleotide excision repair factor XPC enhances DNA damage-induced apoptosis by downregulating the antiapoptotic short isoform of caspase-2. Cancer Res.

[15] Jeanes A, Gottardi CJ, Yap AS (2008). Cadherins and cancer: how does cadherin dysfunction promote tumor progression?. Oncogene.

[16] Onder TT, Gupta PB, Mani SA, Yang J, Lander ES, Weinberg RA (2008). Loss of E-cadherin promotes metastasis via multiple downstream transcriptional pathways. Cancer Res.

[17] Birchmeier W, Behrens J (1994). Cadherin expression in carcinomas: role in the formation of cell junctions and the prevention of invasiveness. Biochim Biophys Acta.

[18] Thiery JP (2002). Epithelial-mesenchymal transitions in tumour progression. Nat Rev Cancer.

[19] Yang YL, Chen MW, Xian L (2014). Prognostic and clinicopathological significance of downregulated E-cadherin expression in patients with non-small cell lung cancer (NSCLC): a meta-analysis. PLoS One.

[20] Yan HB, Wang XF, Zhang Q, Tang ZQ, Jiang YH, Fan HZ, Sun YH, Yang PY, Liu F (2014). Reduced expression of the chromatin remodeling gene ARID1A enhances gastric cancer cell migration and invasion via downregulation of E-cadherin transcription. Carcinogenesis.

[21] Tripathi V, Popescu NC, Zimonjic DB (2014). DLC1 induces expression of E-cadherin in prostate cancer cells through Rho pathway and suppresses invasion. Oncogene.

[22] Wu YH, Tsai Chang JH, Cheng YW, Wu TC, Chen CY, Lee H (2007). Xeroderma pigmentosum group C gene expression is predominantly regulated by promoter hypermethylation and contributes to p53 mutation in lung cancers. Oncogene.

[23] Stockinger A, Eger A, Wolf J, Beug H, Foisner R (2001). E-cadherin regulates cell growth by modulating proliferation-dependent beta-catenin transcriptional activity. J Cell Biol.

[24] Pecina-Slaus N (2003). Tumor suppressor gene E-cadherin and its role in normal and malignant cells. Cancer Cell Int.

[25] Moreno-Bueno G, Portillo F, Cano A (2008). Transcriptional regulation of cell polarity in EMT and cancer. Oncogene.

[26] Peinado H, Ballestar E, Esteller M, Cano A (2004). Snail mediates E-cadherin repression by the recruitment of the Sin3A/histone deacetylase 1 (HDAC1)/HDAC2 complex. Mol Cell Biol.

[27] Bolos V, Peinado H, Perez-Moreno MA, Fraga MF, Esteller M, Cano A (2003). The transcription factor Slug represses E-cadherin expression and induces epithelial to mesenchymal transitions: a comparison with Snail and E47 repressors. J Cell Sci.

[28] Singh AB, Sharma A, Smith JJ, Krishnan M, Chen X, Eschrich S, Washington MK, Yeatman TJ, Beauchamp RD, Dhawan P (2011). Claudin-1 up-regulates the repressor ZEB-1 to inhibit E-cadherin expression in colon cancer cells. Gastroenterology.

[29] Lau MT, Leung PC (2012). The PI3K/Akt/mTOR signaling pathway mediates insulin-like growth factor 1-induced E-cadherin down-regulation and cell proliferation in ovarian cancer cells. Cancer Lett.

[30] Hsu YL, Hou MF, Kuo PL, Huang YF, Tsai EM (2013). Breast tumor-associated osteoblast-derived CXCL5 increases cancer progression by ERK/MSK1/Elk-1/snail signaling pathway. Oncogene.

[31] Tang D, Wu D, Hirao A, Lahti JM, Liu L, Mazza B, Kidd VJ, Mak TW, Ingram AJ (2002). ERK activation mediates cell cycle arrest and apoptosis after DNA damage independently of p53. J Biol Chem.

[32] Rezvani HR, Kim AL, Rossignol R, Ali N, Daly M, Mahfouf W, Bellance N, Taieb A, de VH, Mazurier F, Bickers DR (2011). XPC silencing in normal human keratinocytes triggers metabolic alterations that drive the formation of squamous cell carcinomas. J Clin Invest.

[33] Han W, Soltani K, Ming M, He YY (2012). Deregulation of XPC and CypA by cyclosporin A: an immunosuppression-independent mechanism of skin carcinogenesis. Cancer Prev Res (Phila).

[34] Wu YH, Cheng YW, Chang JT, Wu TC, Chen CY, Lee H (2007). Reduced XPC messenger RNA level may predict a poor outcome of patients with nonsmall cell lung cancer. Cancer.

[35] Nollet F, Berx G, van RF (1999). The role of the E-cadherin/catenin adhesion complex in the development and progression of cancer. Mol Cell Biol Res Commun.

[36] Yanagisawa M, Anastasiadis PZ (2006). p120 catenin is essential for mesenchymal cadherin-mediated regulation of cell motility and invasiveness. J Cell Biol.

[37] Soto E, Yanagisawa M, Marlow LA, Copland JA, Perez EA, Anastasiadis PZ (2008). p120 catenin induces opposing effects on tumor cell growth depending on E-cadherin expression. J Cell Biol.

[38] Tsukamoto T, Nigam SK (1999). Cell-cell dissociation upon epithelial cell scattering requires a step mediated by the proteasome. J Biol Chem.

[39] Wijnhoven BP, Dinjens WN, Pignatelli M (2000). E-cadherin-catenin cell-cell adhesion complex and human cancer. Br J Surg.

[40] Ma L, Young J, Prabhala H, Pan E, Mestdagh P, Muth D, Teruya-Feldstein J, Reinhardt F, Onder TT, Valastyan S, Westermann F, Speleman F, Vandesompele J, Weinberg RA (2010). miR-9, a MYC/MYCN-activated microRNA, regulates E-cadherin and cancer metastasis. Nat Cell Biol.

[41] Hsu YL, Huang MS, Yang CJ, Hung JY, Wu LY, Kuo PL (2011). Lung tumor-associated osteoblast-derived bone morphogenetic protein-2 increased epithelial-to-mesenchymal transition of cancer by Runx2/Snail signaling pathway. J Biol Chem.

[42] Zhang HM, Li L, Papadopoulou N, Hodgson G, Evans E, Galbraith M, Dear M, Vougier S, Saxton J, Shaw PE (2008). Mitogen-induced recruitment of ERK and MSK to SRE promoter complexes by ternary complex factor Elk-1. Nucleic Acids Res.

[43] Chen H, Zhu G, Li Y, Padia RN, Dong Z, Pan ZK, Liu K, Huang S (2009). Extracellular signal-regulated kinase signaling pathway regulates breast cancer cell migration by maintaining slug expression. Cancer Res.

[44] Marchetti A, Colletti M, Cozzolino AM, Steindler C, Lunadei M, Mancone C, Tripodi M (2008). ERK5/MAPK is activated by TGFbeta in hepatocytes and required for the GSK-3beta-mediated Snail protein stabilization. Cell Signal.

[45] Wei F, Yan J, Tang D (2011). Extracellular signal-regulated kinases modulate DNA damage response - a contributing factor to using MEK inhibitors in cancer therapy. Curr Med Chem.

[46] Fong YW, Inouye C, Yamaguchi T, Cattoglio C, Grubisic I, Tjian R (2011). A DNA repair complex functions as an Oct4/Sox2 coactivator in embryonic stem cells. Cell.

[47] Wang QE, Zhu Q, Wani G, El-Mahdy MA, Li J, Wani AA (2005). DNA repair factor XPC is modified by SUMO-1 and ubiquitin following UV irradiation. Nucleic Acids Res.

[48] Han C, Zhao R, Liu X, Srivastava A, Gong L, Mao H, Qu M, Zhao W, Yu J, Wang QE (2014). DDB2 suppresses tumorigenicity by limiting the cancer stem cell population in ovarian cancer. Mol Cancer Res.

[49] Gottardi CJ, Wong E, Gumbiner BM (2001). E-cadherin suppresses cellular transformation by inhibiting beta-catenin signaling in an adhesion-independent manner. J Cell Biol.

