# Supplementary material for: XPC inhibits NSCLC cell proliferation and migration by enhancing E-Cadherin expression
Source: Oncotarget. 2015 Mar 12;6(12):10060–72. doi: 10.18632/oncotarget.3542 (PMC4496340; doi:10.18632/oncotarget.3542)
Supplement: Supplementary file 1 [file oncotarget-06-10060-s001.pdf]

XPC inhibits NSCLC cell proliferation and migration by enhancing E-Cadherin expression

Supplementary Material

Analysis of publicly available datasets

To analyze the effect of *XPC* expression on prognostic of lung cancer patients, we generated Kaplan-Meier survival curve of NSCLC patients with low or high expression of *XPC* by using Kaplan-Meier Plotter (www.kmplot.com/analysis) (Supplementary Figure 1A-D). Specifically, select “Lung cancer” at the upper-right corner of the home page of www.kmplot.com/analysis, input “XPC” into Affy id/Gene symbol (209375_at), click “Auto select best cutoff”, then click “Draw Kaplan-Meier plot”. The patients were automatically split to low XPC and high XPC groups according to the *XPC* expression values determined by microarray.

To analyze the relationship between *XPC* mRNA and E-Cadherin protein levels in lung adenocarcinomas, we retrieved and analyzed the data from TCGA dataset by using a tool in www.cbioportal.org. Specifically, select “Query” on the home page of the website www.cbioportal.org, selects “Lung Adenocarcinoma (TCGA, Provisional)” from Select Cancer Study. In the “Select Genomic Profiles”, select “mRNA Expression z-Score (RNA Seq V2 RSEM)” and “protein/phosphoprotein level (RPPA)”. In “Enter Gene set”, input “XPC: EXP < 0”, then click “Submit”. On the next page, click “Protein Change” tab, then change Antibody Type to “protein_level”, click CDH1 (encoding E-Cadherin), the corresponding figure will show.

To analyze the relationship between *XPC* and *Snai1, Snai2, or Zeb1* mRNA levels in lung adenocarcinomas, we obtained the data from TCGA, Nature 2014 by using www.cbioportal.org. Specifically, on the home page of the website, select “download data”, then, select “Lung Adenocarcinoma (TCGA, Nature 2014)”, click “mRNA expression Z-score (microarray)” from Select Genomic Profiles, and enter gene set “XPC, SNAI1, SNAI2, ZEB1”, select “Transpose data matrix”. Click “Submit”, the *XPC, SNAI1, SNAI2, and ZEB1* mRNA Z-scores of 230 cases will appear. The correlation between XPC and other genes Z-scores was then analyzed by Pearson correlation and plotted using Minitab.

Wound-healing assay

Cells were cultured in 6-well plates until full confluence. Cell monolayer was carefully scratched using a 200-μl sterile pipette tip and washed twice with fresh medium. Cells were cultured in the presence of 5μg/ml of mitomycin C to inhibit cell proliferation. The wound edges were photographed under an inverted-phase microscope after 24 h, and measured.

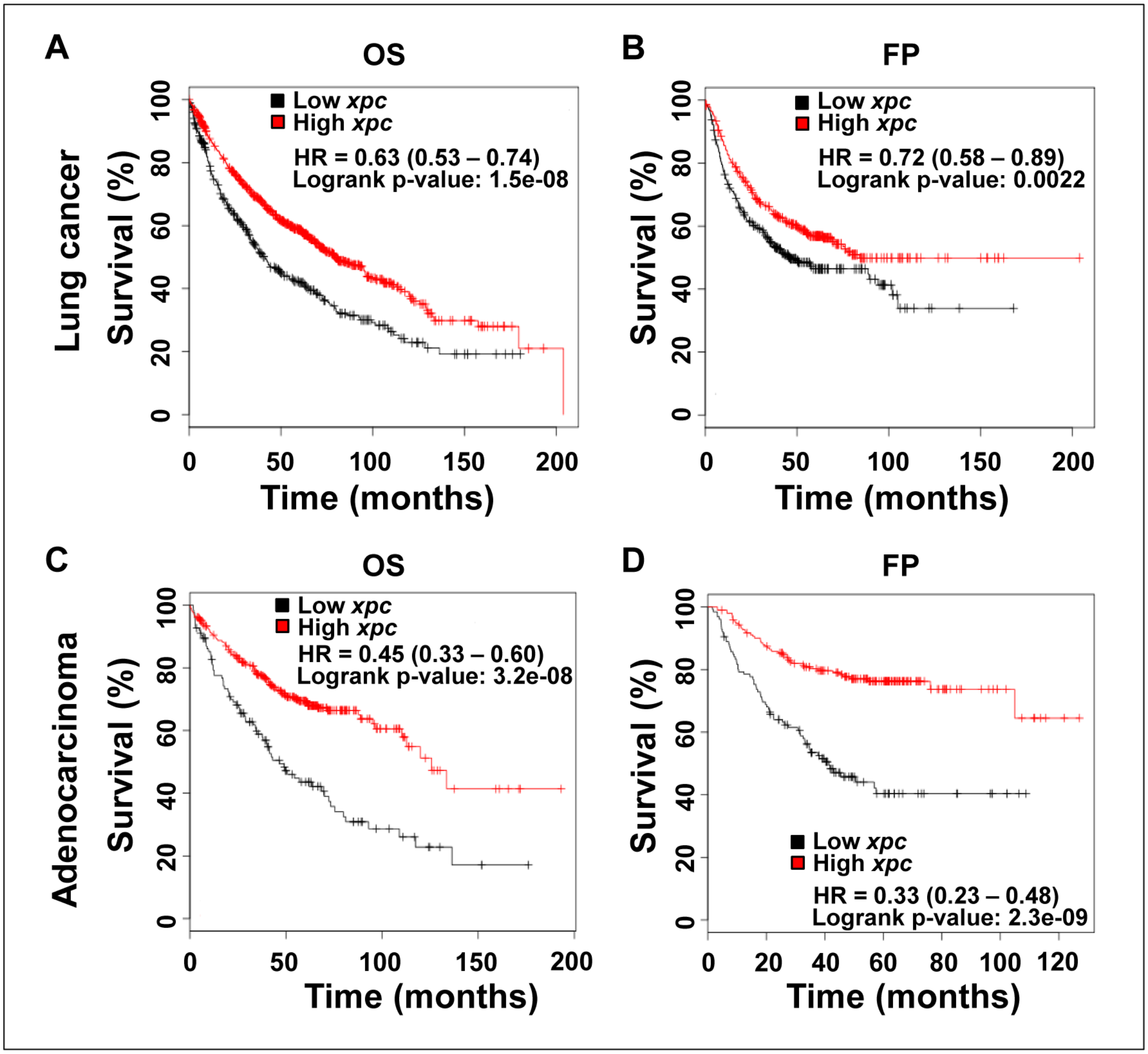

Supplementary Figure S1: Prognostic significance of XPC in lung cancer. (**A**,**B**) The effect of *XPC* mRNA expression level on the overall survival (**A**) and progression free survival (**B**) in 1,432 lung cancer patients was analyzed and the Kaplan-Meier plots were generated by Kaplan-Meier Plotter (http://www.kmplot.com). (**C**,**D**) The effect of XPC mRNA expression level on the overall survival (**A**) and progression free survival (**B**) in 865 lung adenocarcinoma patients was analyzed and the Kaplan-Meier plots were generated by Kaplan-Meier Plotter (http://www.kmplot.com).

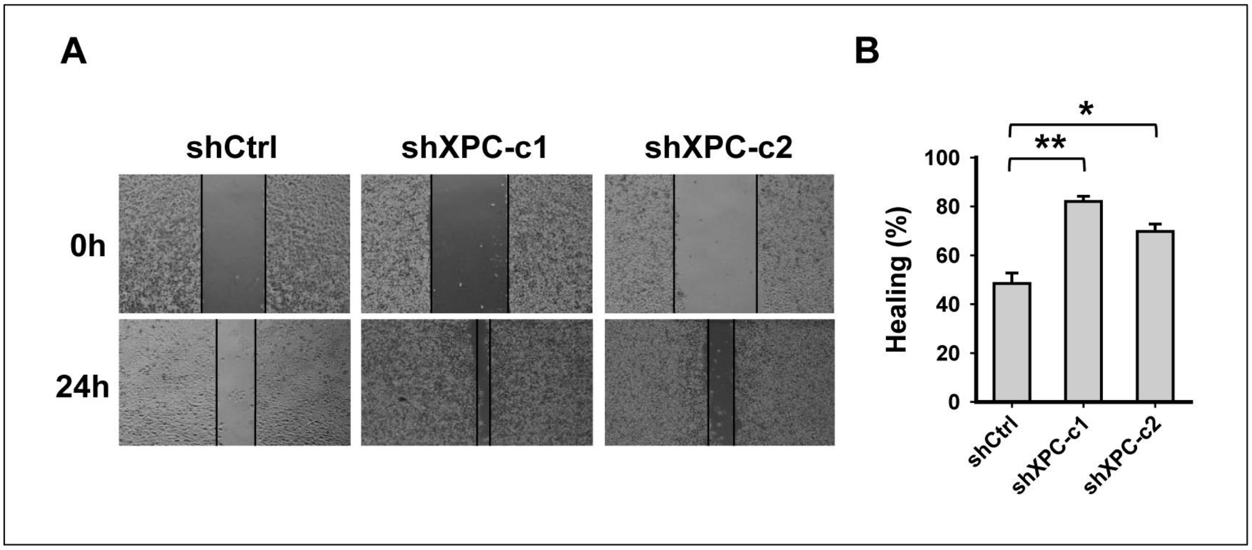

Supplementary Figure S2: XPC knockdown increased NSCLC cell migration *in vitro*. (**A**) Cell motility was determined by scratch wound healing assays. (**B**) Mobility rate histograms of each group. n=3, *, *P*<0.05; **, *P*<0.01.

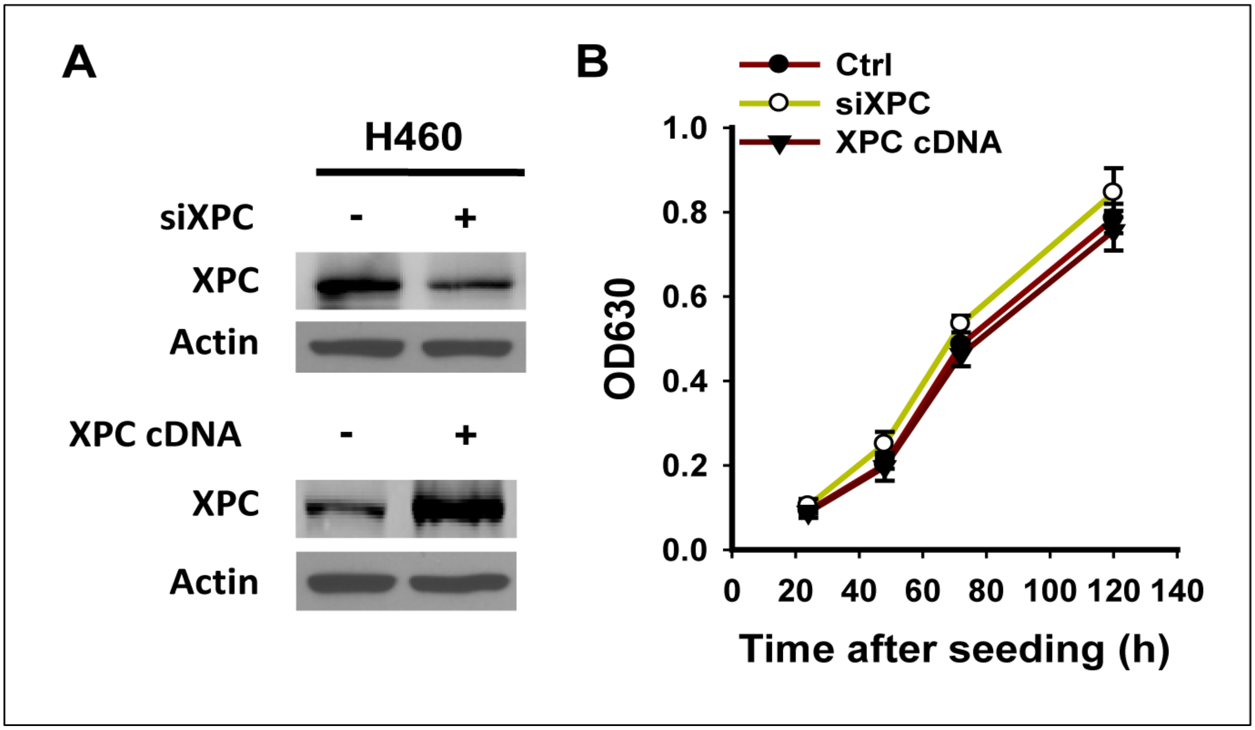

Supplementary Figure S3: XPC modulation did not affect cell growth of NSCLC cell line H460. H460 cells were transfected with either XPC siRNA to knock down XPC expression, or XPC expressing plasmids to enhance cellular XPC protein level. (**A**) Western blotting analysis confirmed downregulation of XPC in siXPC-transfected cells and overexpression of XPC in XPC cDNA-transfected cells. (**B**) Cell growth dynamics were determined. n = 5, bar: SD.

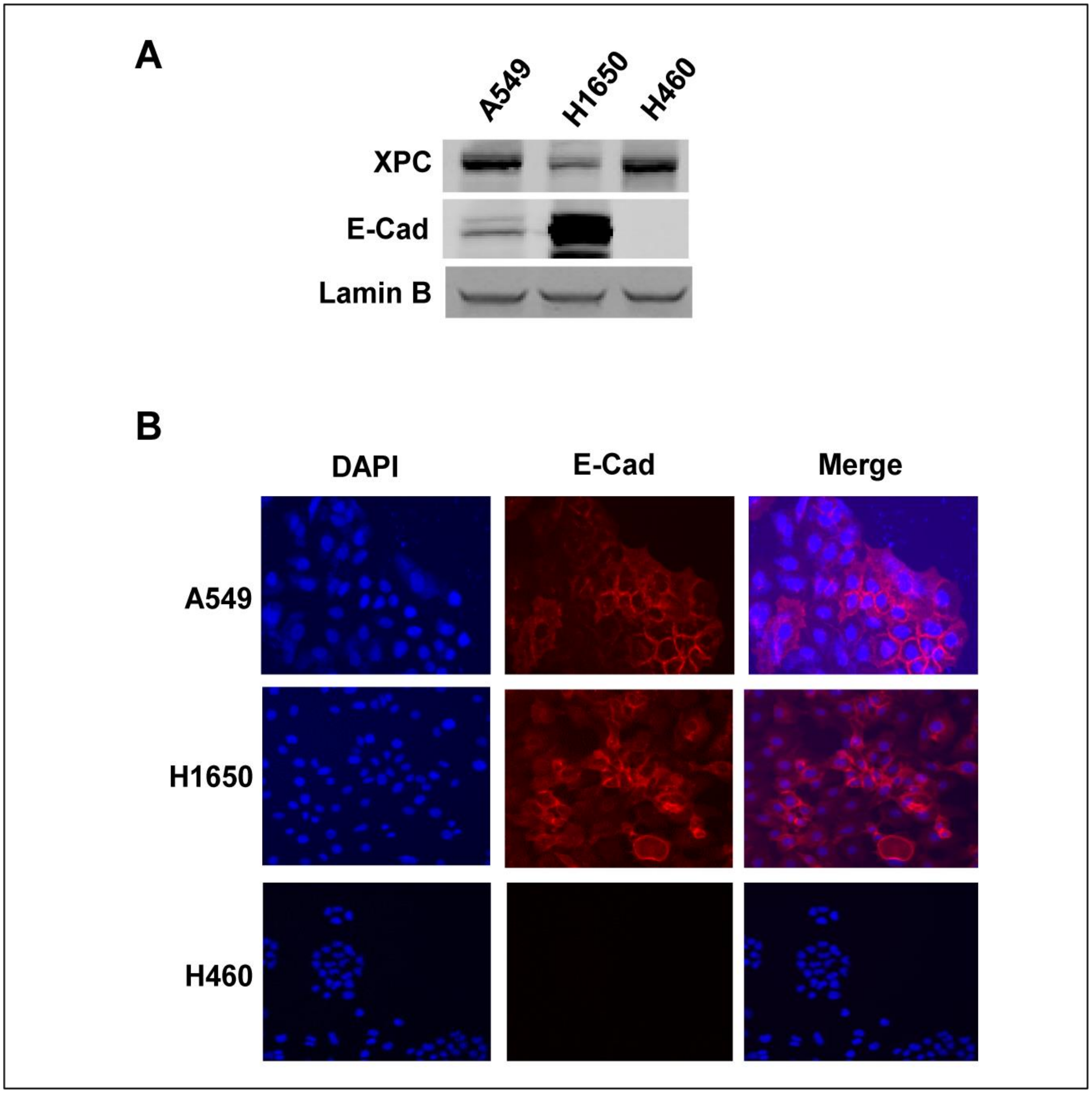

Supplementary Figure S4: The expression level of E-Cadherin in various NSCLC cell lines. The expression of E-Cadherin in A549, H1650 and H460 cells were determined using either immunoblotting (**A**) or immunofluorescence (**B**).

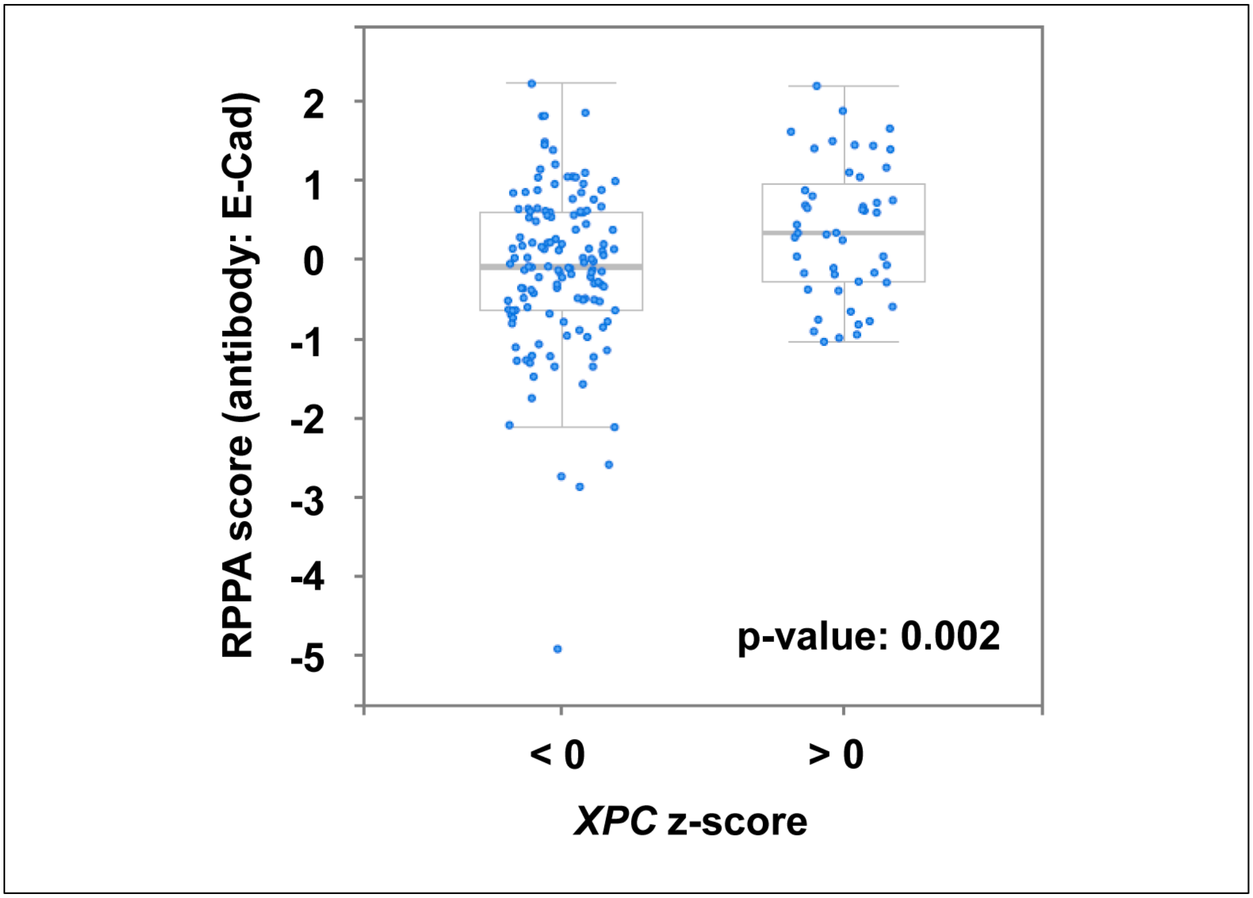

Supplementary Figure S5: The relationship between *XPC* mRNA level and E-Cadherin protein level in human lung adenocarcinoma. The *XPC* mRNA and E-Cadherin protein expression data of human lung adenocarcinoma were retrieved from TCGA dataset. The patients were divided into two groups based on the *XPC* mRNA z-score, and the E-Cadherin protein level detected by Reverse phase protein array (RPPA) was plotted.

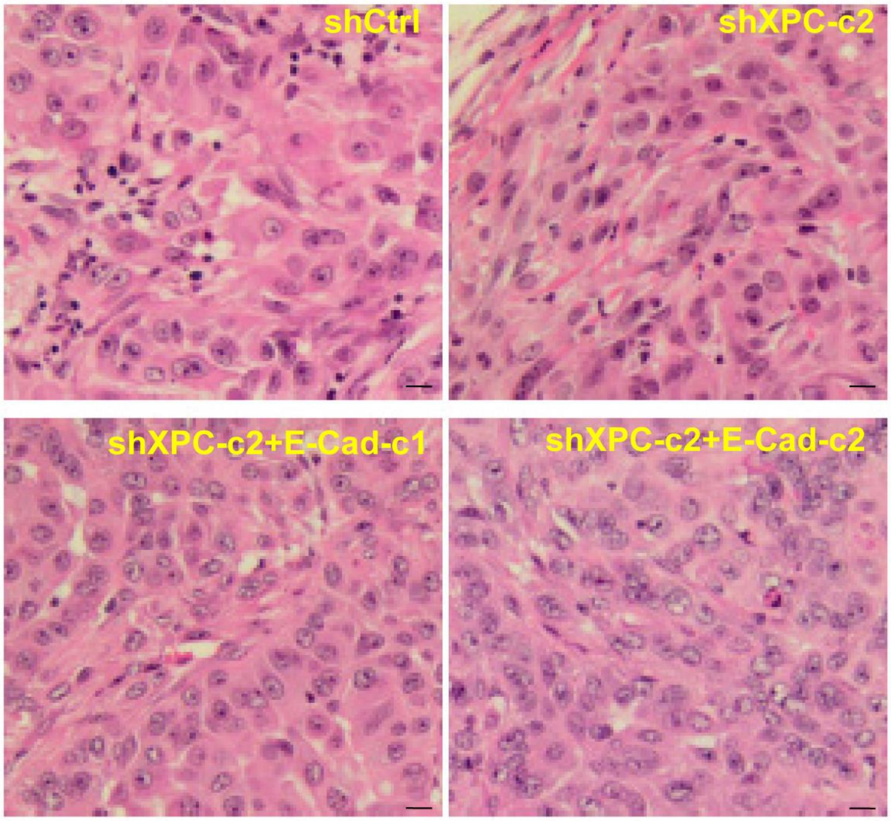

Supplementary Figure S6: Histopathological examination of Xenografts. Xenografts were formalin-fixed, paraffin-embedded, sliced and stained with H&E. Representative images of each group are presented (scale bar: 50 µm).

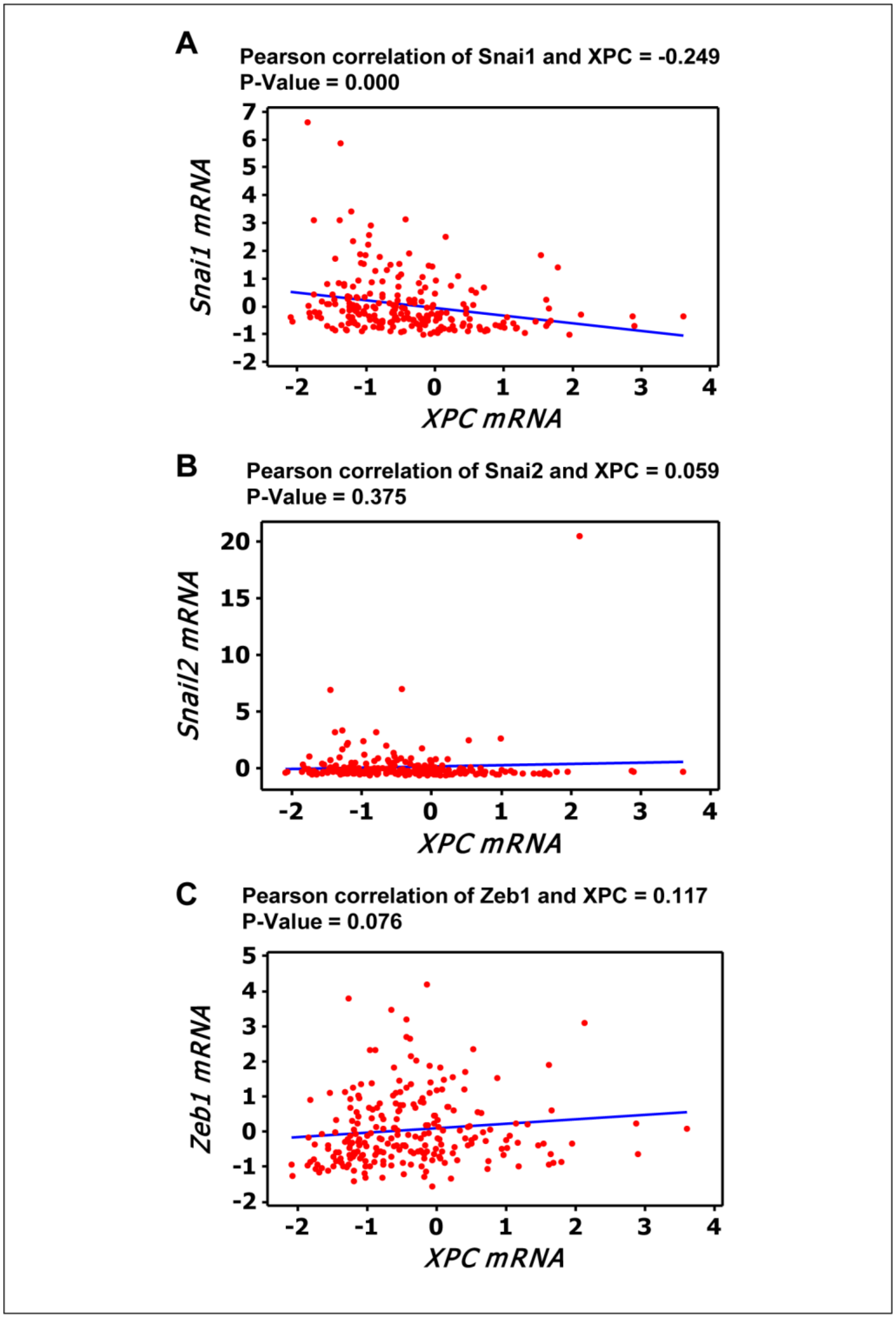

Supplementary Figure S7: The relationship between *XPC* and various EMT transcription factors mRNA expression levels in human lung adenocarcinoma. The *XPC*, *Snai1*, *Snai2*, and *Zeb1* mRNA z-scores were retrieved from TCGA dataset by using www.cbioportal.org. The correlation between *XPC* and other genes mRNA z-scores was then analyzed by Pearson correlation and plotted using Minitab.

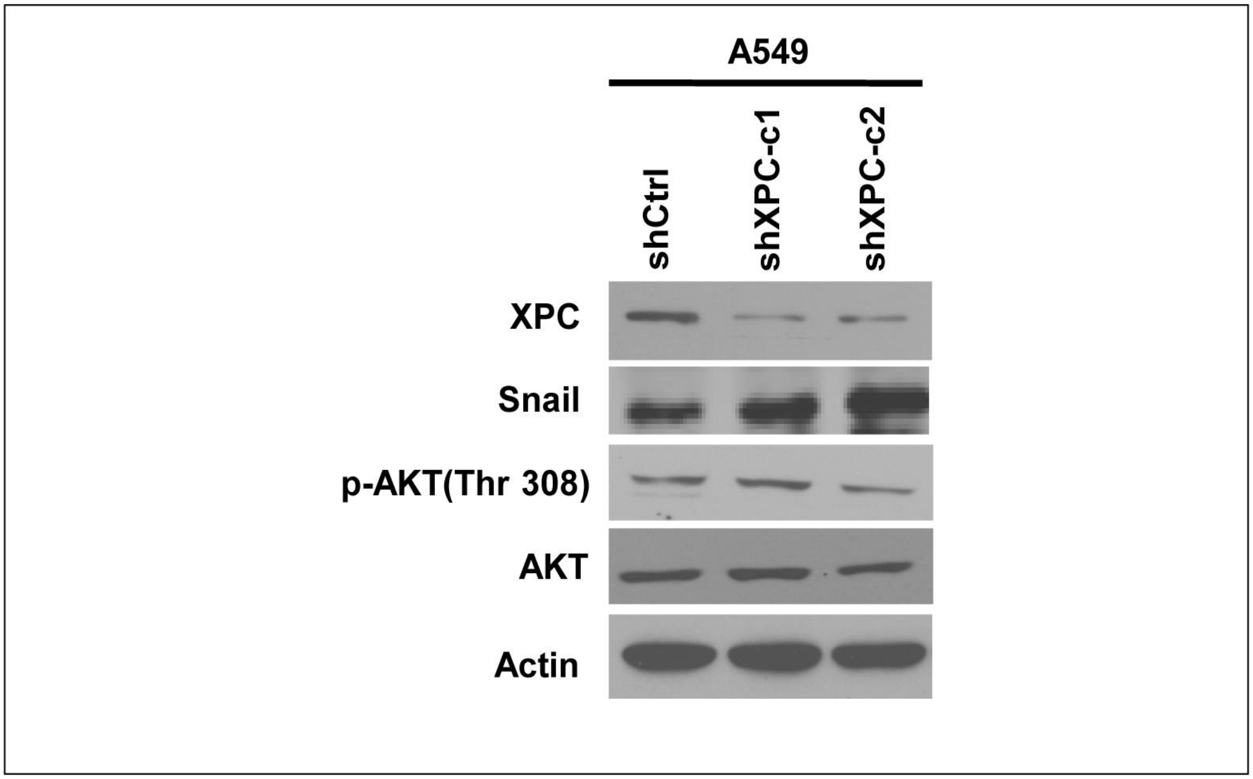

Supplementary Figure S8: Snail expression is enhanced in A549 cells with stable XPC downregulation. Immunoblotting was conducted to analyze the protein level of XPC, Snail, phosphorylated AKT, AKT, and Actin in A549 cells and two clones of shXPC stably transfected A549 cells.

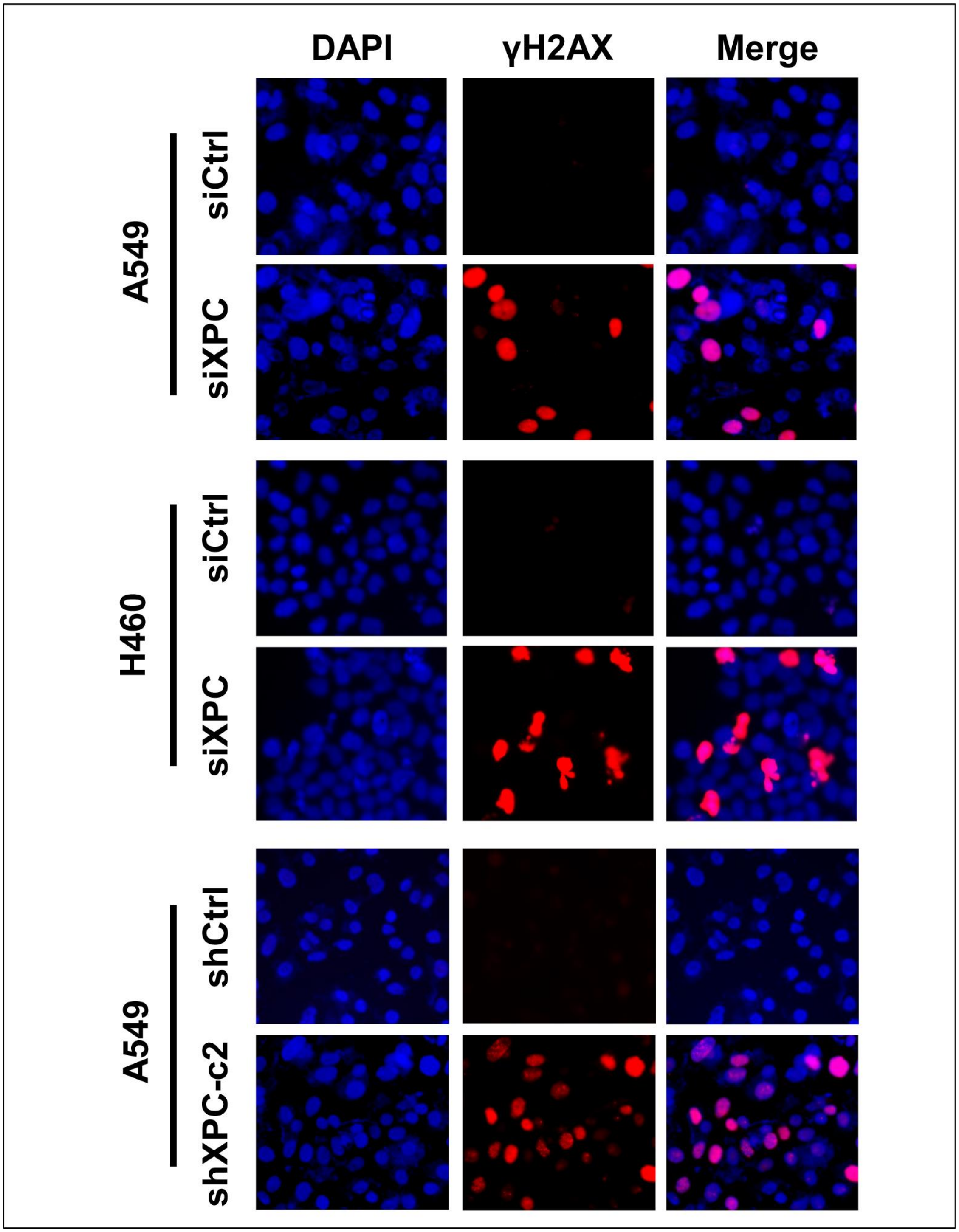

Figure S9: Downregulation of XPC increases endogenous DNA damage. Immunofluorescent staining was conducted to visualize γH2AX-positive cells in A549 and H460 cells transiently transfected with either control or XPC siRNA, as well as A549 cells stably transfected with shXPC. Representative immunofluorescence images are presented.
